# The chiropractic profession in Denmark 2010–2014: a descriptive report

**DOI:** 10.1186/s12998-015-0072-9

**Published:** 2015-09-14

**Authors:** Orla Lund Nielsen, Alice Kongsted, Henrik Wulff Christensen

**Affiliations:** The Nordic Institute of Chiropractic and Clinical Biomechanics, Campusvej 55, DK-5230 Odense M, Denmark; Department of Sports Science and Clinical Biomechanics, University of Southern Denmark, Campusvej 55, 5230 Odense M, Denmark

**Keywords:** Chiropractic, Clinical practice pattern, PrimaryHealth Care, Denmark

## Abstract

**Background:**

The chiropractic profession has been well established in Denmark for several decades with state authorization, partial reimbursement by the state and a formal academic education. Biennial systematic data collections among all chiropractors and clinics have been performed since 2010 in order to provide exact information on the profession to The Danish Chiropractic Association (DCA). It is the aim of this study to outline the major characteristics and developments of the chiropractic profession in Denmark to make this information accessible to other stakeholders, domestic as well as foreign.

**Methods:**

Using contact information from the DCA, two questionnaires were distributed electronically to all individual members of the association actively working as chiropractors and all clinics respectively in 2010, 2012 and 2014. The questions asked were developed for this specific survey.

**Results:**

Response rates varied between 59 and 78 % for the clinic questionnaires and 75 to 86 % for the individual questionnaires. Almost half the Danish chiropractors were educated in Denmark and a small majority was female. The average Danish chiropractor of 2014 was 44 years old, graduated 17 years earlier, and worked full time in a primary care clinic with at least one colleague. Half the chiropractors spent more than 20 h a year on continued professional development. Danish chiropractic clinics had a median of 3 treatment rooms, most had digital X-ray equipment, around 6 out of 10 had exercise facilities, and 1 out of 4 employed a physiotherapist. Three out of 4 clinics employed a secretary, too. The average duration of a consultation was 40 min for a new patient and 13 min for a follow-up consultation. Virtually all Danish chiropractors working in the primary sector made use of manipulation as one of their treatment modalities.

**Conclusion:**

This is the first study to describe the state and latest development of the chiropractic profession in Denmark using repeated surveys. Displaying various characteristics of both clinics and individual chiropractors, the image emerging is one of a stable profession where rapid or drastic changes are not taking place over short intervals of time.

## Background

### Organisation of chiropractic in Denmark

The first chiropractor in Denmark started practising in 1920. Since then a profession has emerged and it has been an acknowledged and accepted part of the official Danish health care system for several decades by now. Although only to a limited degree, partial reimbursement of expenses for chiropractic care was acceded to in 1978 by the Danish government, and in 1992 a state authorization for practicing as a chiropractor was introduced as the final step towards full recognition of the profession [1–3].

A 5-year academic chiropractic education was introduced in 1994 at what is now called The University of Southern Denmark (SDU) in Odense, and since 1999 when the first students completed their education approximately 300 Danish chiropractors (by 2014) have joined the profession after graduating at SDU largely supplanting United Kingdom and USA as the most common place of education [1].

The Danish Chiropractors’ Association (DCA) was established in 1925. Since then, it has been the sole chiropractic professional organisation in Denmark except for a two-year period in the late 1940s. Affiliation to the association is a requirement for receiving reimbursement from the national health insurance because the DCA negotiates the agreement with the Danish health authorities (The Regions’ Board for Wages and Tariffs) on behalf of the profession. Negotiations take place every three years and includes agreements on payment to chiropractors, regulations related to the profession, and which developmental initiatives the profession should take on. State reimbursement amounts to approximately 20 % of the treatment fee.

The association organizes clinic owners, chiropractic employees in both the primary and the secondary sector, researchers and university lecturers with a chiropractic background, and retired chiropractors as non-active members.

Patients either self-refer to chiropractors or are advised about chiropractic care by a general practitioner. There is no formal referral from general practice and patient costs are the same whether self-referring or being advised to seek chiropractic care by a general practitioner.

In 1987, the DCA established The Foundation for Chiropractic Research and Post Graduate Education in collaboration with The Regions’ Board for Wages and Tariffs (RBWT). Among the objectives of the foundation is to provide financially support for research projects, international stipends, and quality development projects within the field of clinical biomechanics as well as supporting continued professional development. One third of the foundation’s yearly income is provided by RBWT while two thirds is drawn directly from the state reimbursements to the clinics.

### Study background

In 2010 a desire to investigate the major characteristics of the now fairly well established profession and to initiate an on-going monitoring of its further development caused the launching of the first systematic data collection on the chiropractic profession in Denmark. The main purpose of the initiative was to provide comprehensive information to The Danish Chiropractors’ Association to help the organisation in its effort to refine and develop the profession even further. However, we regard this information to be relevant to chiropractors, healthcare professionals, officials, and chiropractic associations in other countries too.

Over the past decade, similar descriptive reports on the chiropractic profession have been issued from a number of our neighbouring countries, mainly displaying characteristics of the individual country at a specific time. A majority of these deal with chiropractic communities somewhat smaller than the Danish profession and presents comparatively few responders (Germany *n* = 49 [4], Finland *n* = 44 [5], Belgium *n* = 80 [6], The Netherlands *n* = 113 [7]), and one of them deals with chiropractors’ beliefs and professional opinions rather than a quantitative description of the profession [8]. A Norwegian study very similar to the Danish survey and actually based on the Danish questionnaires obtained 320 responses from individual chiropractors in 2011 [9], and a study from Switzerland based on 183 participants explicitly aimed at comparing data with data from other countries [10]. Finally a comprehensive American analysis included data from a number of surveys performed in USA over a period of nearly 20 years, the latest of which is from 2009 and had close to 2.300 responders [11].

The primary objective of this report is to describe the chiropractic profession in Denmark in 2014, tentatively outlining developments since 2009 when possible. However, due to the short intervals between the surveys, some data will only be reported for 2014.

Though it is not a main objective of this study to present an exhaustive comparison between countries, in cases where conspicuous similarities or differences call for attention details from previously published reports will be taken into account in the discussion.

## Method

Between 2010 and 2014, three cross-sectional data collections were carried out among Danish chiropractors using contact information obtained from the DCA. Two separate questionnaires were distributed electronically in each survey: one directed at all individual chiropractors who were registered members of the DCA and actively working as chiropractors at the time of initiating the data collection, and one addressed to each separate physical clinic. Hence one owner from each clinic was required to answer both questionnaires.

Designing the project, data collection and analysis were anchored at the Nordic Institute of Chiropractic and Clinical Biomechanics (NIKKB). Mails with a short introduction to the survey signed by a senior researcher at NIKKB and a link to the questionnaire itself were distributed via the licensed online survey database SurveyXact [12]. Analysis was performed in STATA14 [13]. Non-responders and responders who had only filled out part of the questionnaires automatically received reminders one and two weeks later. One or two further reminders were sent manually to non-responders and after approximately five weeks the data collection was closed.

The questions asked were developed for this specific survey and were not previously validated. *Years since education, Physical size of the clinic, Number of treatment rooms, No. of chiropractors* and all questions concerning the duration of consultations were collected as continuous variables. Answer categories to the other questions appear from the tables. Average age was calculated on the basis of 10-year age groups.

According to Danish legislation stipulated in Act on Research Ethics Review of Health Research Projects, section 14, subsection 1–2, approval from The Health Research Ethics Committee System in Denmark was not required as the questionnaires do not ask for personal information and all data were collected anonymously [14].

## Results

### Response rates and geographical representativeness

The response rates for the surveys are shown in Table 1. Some of the questionnaires were only partially completed, which accounts for the “missing” category in the majority of the tables.

**Table 1:** Response rates

*Questionnaire type*	2010	2012	2014
Clinic questionnaire	59 % (143/244)	77 % (193/250)	78 % (201/258)
Individual questionnaire	75 % (393/524)	86 % (469/547)	82 % (454/551)

**Fig. 1:**
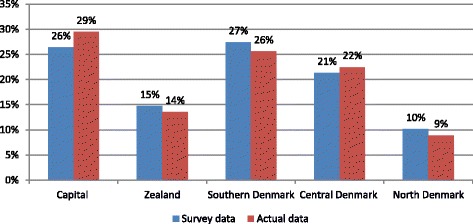
Regional distribution of clinics in Denmark 2014. Survey data compared to the actual distribution of chiropractic clinics in Denmark on the five Regions

Assessment of representativeness could be based on the actual regional distribution of the clinics in 2014 known from the DCA mailing list. A comparison with responses to the clinic questionnaires that same year (Fig. 1) showed the Capital Region of Denmark (Region Hovedstaden) and the Central Denmark Region (Region Midtjylland) to be slightly underrepresented in the survey. A similar pattern was seen in 2012.

### Demographic data

In the 2014 survey, 252 of the 454 (56 %) respondents were female representing a slight increase compared to the earlier surveys (Table 2). Almost half the respondents were 40 years old or younger in 2010 while only 40 % of the responders belonged to this age group in 2014. In the same period of time the percentage of chiropractors aged more than 50 years increased from 23 to 30 %. The calculated average age increased from 41.7 years in 2010 over 42.6 in 2012 to 44.2 years in 2014.

**Table 2:** Demographic data

*Gender*	2010 (*n* = 393)	2012 (*n* = 469)	2014 (*n* = 454)
Male	46 %	44 %	43 %
Female	54 %	54 %	56 %
Missing	0 %	2 %	1 %
*Age group*	2010 (*n* = 393)	2012 (*n* = 469)	2014 (*n* = 454)
21–30 years	12 %	9 %	8 %
31–40 years	39 %	36 %	32 %
41–50 years	26 %	31 %	29 %
51–60 years	18 %	18 %	22 %
61–70 years	5 %	6 %	8 %
>70 years	<1 %	<1 %	<1 %
*Place of graduation*	2010 (*n* = 393)	2012 (*n* = 469)	2014 (*n* = 454)
Denmark	47 %	47 %	46 %
United Kingdom	19 %	20 %	19 %
USA	31 %	29 %	29 %
Canada	2 %	3 %	2 %
Other countries	0 %	1 %	1 %
Missing	1 %	1 %	2 %
*Years since graduation*	2010 (*n* = 393)	2012 (*n* = 469)	2014 (*n* = 454)
0–10 years	42 %	35 %	28 %
11–20 years	19 %	24 %	24 %
21–30 years	24 %	25 %	24 %
>30 years	15 %	14 %	13 %
Missing	0 %	2 %	10 %

Denmark was the most common place of education with a little less than half the responders having graduated here, some 20 % in the United Kingdom and 30 % in the USA. Only small differences could be seen from 2010 to 2014.

In line with a smaller proportion of chiropractors belonging to the youngest age groups, the proportion of responders having graduated less than a decade ago steadily decreased over the study period (Table 2). However, the last survey suffered from some missing values on this particular topic.

### Clinic characteristics

The proportion of clinics covered by the agreement with The Danish health authorities ensuring partial reimbursement was 86 % (173/201) in the 2014 survey.

The average Danish chiropractic clinic had 4 treatment rooms in 2014 and a size of 223 m^2^ which is roughly the same as in the previous years (Table 3). The proportion of medium sized clinics with 3–5 treatment rooms rose slightly to nearly half the clinics in 2014. Very large clinics with more than 10 treatment rooms made up 2 % equalling 5 clinics in 2014. The previous surveys listed 3 and 2 such clinics respectively.

**Table 3:** Physical characteristics of the clinic

*Physical size of the clinic*	2010 (*n* = 143)	2012 (*n* = 193)	2014 (*n* = 201)
Average size (m^2^)	226 m^2^	216 m^2^	223 m^2^
<100 m^2^	10 %	10 %	12 %
100–199 m^2^	39 %	44 %	37 %
200–299 m^2^	22 %	25 %	23 %
300–399 m^2^	10 %	8 %	12 %
≥400 m^2^	10 %	9 %	10 %
Missing	9 %	4 %	5 %
*Number of treatment rooms*	2010 (*n* = 143)	2012 (*n* = 193)	2014 (*n* = 201)
1–2	32 %	37 %	35 %
3–5	43 %	44 %	48 %
6–10	15 %	15 %	11 %
>10	2 %	1 %	2 %
Missing	8 %	4 %	3 %
*Exercise facilities* ^a^	2010 (*n* = 143)	2012 (*n* = 193)	2014 (*n* = 201)
Separate exercise room at the clinic	31 %	22 %	29 %
Gym machines	16 %	15 %	15 %
Movable exercise equipment	52 %	47 %	51 %
Shower	14 %	9 %	12 %
No facilities	39 %	48 %	41 %
Missing	8 %	4 %	4 %
Average size of exercise rooms (m^2^)	113 m^2^	122 m^2^	123 m^2^
*Diagnostic imaging facilities* ^a^	2010 (*n* = 143)	2012 (*n* = 193)	2014 (*n* = 201)
Digital X-ray equipment	57 %	63 %	70 %
Analogue X-ray equiment	25 %	15 %	5 %
Ultrasound	18 %	19 %	17 %
MR imaging	7 %	1 %	1 %
None	9 %	13 %	17 %
Missing	10 %	5 %	4 %

^a^The possibility to give more than one answer was explicitly stated in the questionnaire

Approximately half the clinics offered the use of movable exercise equipment to their patients. The proportion of clinics with a separate exercise room located on the same address remained relatively stable around 30 % (Table 3).

The proportion of clinics with digital X-ray equipment increased across the survey time points at the same time as the prevalence of analogue X-ray equipment went down. Proportions with diagnostic ultrasound varied little across surveys (Table 3).

### Staffing

Clinics manned by one chiropractor only accounted for little more than one third of the total number (Table 4, no information available for 2010). One out of twenty clinics employed five chiropractors or more.

**Table 4:** Staffing

*No. of chiropractors (incl. owners)*	2010 (*n* = 143)	2012 (*n* = 193)	2014 (*n* = 201)
1	Not available	37 %	36 %
2	Not available	26 %	25 %
3	Not available	13 %	19 %
4	Not available	10 %	8 %
≥5	Not available	6 %	5 %
Missing	Not available	8 %	6 %
*Percentage of clinics employing other staff groups than chiropractors*	2010 (*n* = 143)	2012 (*n* = 193)	2014 (*n* = 201)
Secretaries	69 %	79 %	76 %
Physiotherapists	27 %	25 %	25 %
Masseurs	43 %	46 %	48 %
Medical doctors	1 %	1 %	1 %
Other practitioners	19 %	14 %	19 %
No other employees	9 %	7 %	11 %
Missing	9 %	6 %	5 %
*Multidisciplinary setting* ^a^	52 %	55 %	56 %

^a^Understood as the percentage of clinics employing at least one health practitioner (not secretaries) of any kind

A majority of the clinics employed secretaries. Among other health practitioners masseurs were the group most frequently employed, while physiotherapists were engaged in about one fourth of the clinics.

More than half the clinics had some kind of health practitioner other than a chiropractor employed, e.g. masseurs, physiotherapists, acupuncturists, dieticians or medical doctors etc. (Table 4).

### Employment, working hours and continuing professional development

When looking at the chiropractors’ occupational status in general, i.e. both primary and secondary jobs as a whole, practising in the primary sector is by far the most frequent type of employment (Table 5). Almost two thirds of the practitioners are owners. The proportion of chiropractors employed by the health insurance companies decreased across the survey period.

**Table 5:** Employment, working hours and continued professional development (CPD)

*General employment* ^a^	2010 (*n* = 393)	2012 (*n* = 469)	2014 (*n* = 454)
Owner of a clinic	56 %	58 %	58 %
Employed by a private clinic	32 %	32 %	30 %
Employed in the public sector	11 %	10 %	12 %
Employed by health insurance comp.	13 %	7 %	6 %
Other	8 %	4 %	3 %
Missing	1 %	1 %	4 %
*Total working hours pr. week*	2010 (*n* = 393)	2012 (*n* = 469)	2014 (*n* = 454)
More than 37 h	30 %	32 %	31 %
30–37 h	48 %	46 %	46 %
25–29 h	13 %	12 %	12 %
20–24 h	4 %	5 %	4 %
Less than 20 h	2 %	3 %	4 %
Missing	4 %	2 %	3 %
*Hours of CPD pr. year*	2010 (*n* = 388)	2012 (*n* = 469)	2014 (*n* = 454)
0 h	Not available	1 %	5 %
1–10 h	Not available	13 %	21 %
11–20 h	Not available	16 %	22 %
21–30 h	Not available	20 %	18 %
31–40 h	Not available	12 %	11 %
41–50 h	Not available	6 %	6 %
More than 50 h	Not available	20 %	15 %
Missing	Not available	13 %	3 %

^a^The possibility to give more than one answer was explicitly stated in the questionnaire

In terms of each chiropractor’s primary job only (not reported in the tables) as opposed to employment in general, 11 individuals (2 %) did clinical work at a hospital in the secondary sector, another 11 were engaged by a health insurance company, and 16 chiropractors (4 %) were employed in research and/or teaching in 2014 (*n* = 454). In 2014 the respondents were given the opportunity to state administrational tasks or management as their primary function too and 9 individuals (2 %) did so.

Nearly one out of five chiropractors had two or three employments related to the profession (83 in 2014, 89 in 2012 and 72 in 2010). Teaching formed the largest single job function among this group and was done by 35 % in 2014 (34 % in 2012 and 38 % in 2010).

Most Danish chiropractors worked full time or more (Table 5). Lack of employment was rare with six chiropractors (2 %) declaring not being occupied as a chiropractor in 2010 decreasing to four in 2012 and only one chiropractor lacking employment in 2014.

Owners of a clinic tended to work more than their employees. Figures from 2014 showed that 40 % of the clinic owners reported to work more than 37 h a week in total versus 12 % of the chiropractors in the primary sector who were not self-employed. The proportion of owners working 25–29 h and less than 25 h weekly was 10 and 5 % respectively, whereas the corresponding percentages for employees were 15 and 12 %.

Data on the effort dedicated to continued professional development (CPD) by each respondent was only available from the two latest surveys (Table 5). Half the chiropractors reported more than 20 h a year dedicated to CPD in 2014.

### Working in the primary sector

Chiropractors working in a clinic, whether that may be as their primary or secondary job, naturally spent the vast majority of their working capacity on patient consultations. An average of 29 h weekly was dedicated by these chiropractors to the treatment of patients in 2014, and a little less than 4 h and 2 h were spent on administration and communication with other parties of the Danish health care sector respectively.

The duration of the various kinds of consultations seems remarkably constant (Table 6).

**Table 6:** Work in the primary sector

*Minutes allotted to a new patient*	2010 (*n* = 332)	2012 (*n =* 403)	2014 (*n* = 400)
Average	40 min.	40 min.	40 min.
≤15 min.	1 %	0 %	0 %
16–30 min.	33 %	33 %	33 %
31–45 min.	47 %	49 %	52 %
46–60 min.	16 %	15 %	12 %
>60 min	0 %	0 %	1 %
Missing	2 %	3 %	2 %
*Minutes allotted to known patients presenting with a new problem*	2010 (*n* = 332)	2012 (*n* = 403)	2014 (*n =* 400)
Average	25 min.	26 min.	26 min.
≤15 min.	17 %	11 %	12 %
16–30 min.	73 %	77 %	77 %
31–45 min.	7 %	7 %	8 %
46–60 min.	2 %	2 %	1 %
>60 min	0 %	0 %	0 %
Missing	2 %	3 %	2 %
*Minutes allotted follow-up consultations*	2010 (*n* = 332)	2012 (*n* = 403)	2014 (*n* = 400)
Average	13 min.	13 min.	13 min.
≤5 min.	3 %	1 %	2 %
6–10 min.	50 %	49 %	46 %
11–15 min.	37 %	40 %	40 %
16–30 min.	8 %	6 %	9 %
>60 min	0 %	0 %	0 %
Missing	2 %	3 %	3 %
*Which treatment modalities do you make use of?* ^a^	2010 (*n* = 332)	2012 (*n* = 403)	2014 (*n* = 400)
Manipulation	97 %	97 %	98 %
Trigger point	93 %	93 %	90 %
Other soft tissue techniques	86 %	88 %	86 %
Activator	45 %	50 %	48 %
Shockwave	8 %	11 %	13 %
Acupuncture/dry-needling	38 %	40 %	48 %
Exercise instruction	85 %	83 %	81 %
Laser	Not available	Not available	25 %
Missing	2 %	2 %	1 %

^a^The possibility to give more than one answer was explicitly stated in the questionnaire

Virtually all Danish chiropractors working in the primary sector make use of manipulation while trigger point treatment and other soft tissue techniques are offered by 85-90 % and exercise instructions by more than 80 %. The Danish surveys did not ask about the proportion of patients the various treatment modalities were given to. 

## Discussion

### General findings

The average Danish chiropractor of 2014 is 44 years old, graduated 17 years ago and works full time in a primary care clinic with at least one colleague. Danish chiropractic clinics have a median of 3 treatment rooms, most have digital X-ray equipment, around 6 out of 10 have exercise facilities, and 1 out of 4 employs a physiotherapist. The described profile of the Danish chiropractic profession did not undergo large changes from 2010 to 2014.

### Quality of the data

A response rate above 75 % as seen in five out of the six questionnaires is considered satisfactory, and matches or exceeds comparable studies from our neighbouring countries [4–7, 9–11]. Two of the individual questionnaires even obtain response rates of 82 and 86 %. Only a single question in one of the surveys had more than 10 % missing values.

With contact information made available by the only chiropractic association in Denmark, we were able to contact practically all Danish chiropractors. However, we do not know if non-responders to the surveys may represent a group of clinicians with a certain profile.

Negatively affecting the response rate for the clinic questionnaire may be that owners were required to answer both questionnaires, perhaps mistakenly leading them to believe the questionnaire to be already answered when they got to the second one. A similar effect may have been caused by the fact that the data source was physical clinics which implied that a clinic working from several addresses received several questionnaires probably even using the same email address. However, the results, especially from the 2012- and 2014-surveys, can be viewed as confidently reflecting the actual state of the Danish chiropractic profession.

### The Danish chiropractor

Distinguishing the Danish profession most clearly from the Norwegian, Swiss, Belgian, Dutch or Finnish profession is the very high proportion of female practitioners of 56 % compared to 20–36 % in the said countries. With 43 % female chiropractors, the United Kingdom is the only country relatively close to the Danish gender distribution [5–10].

The American survey displays a female proportion changing from 13 % in 1991 to 22 % in 2009 [11]. A certain increase has been taking place in Denmark too over the years covered by the Danish survey, but due to the short time span it shows somewhat less markedly. Future increase in the proportion of female chiropractors is expected with a majority of Danish chiropractor students being females (from 2010–2014 59–67 % of admitted students were females [15]).

In terms of average age the Danish chiropractor seems to be growing older too. From 2010 to 2014 the proportion of chiropractors still in their twenties has decreased from 12 to 8 % while the 61–70 years age group has increased from 5 to 8 %. At the same time the surveys display a decrease in the proportion of responders with a maximum of 10 years since the date of their graduation from 42 % in 2010 over 35 % in 2012 to just 28 % in 2014, but due to a comparatively high number of missing values in the latest survey on this specific issue we are unable to ascertain to what extent the apparent change may be caused by selection bias.

### Chiropractic clinics, the owners and their employees

Significant changes in the size or characteristic of the average Danish chiropractic clinic cannot be detected over the past four years.

Clinics with 3–5 treatment rooms comprise nearly half the total number of clinics in 2014 which may represent a certain tendency towards medium sized clinics.

The prevalence of diagnostic imaging facilities forms the most distinct development in relation to the physical appearance of the clinic. The proportion of clinics with digital X-ray facilities rose from 57 % in 2010 over 63 % in 2012 to 70 % in 2014. Even more significant is the decrease in the prevalence of analogue X-ray equipment in the same period of time from 25 % over 15 to 5 %. Considered together with the increase in the number of clinics reporting to have no such facilities at all from 9 % over 13 to 17 % we see that whereas a growing number of the clinics acquire digital imaging facilities of their own not all the discarded analogue equipment is substituted by digital facilities. Some clinics simply choose to refer their patients to somewhere else for imaging.

Though a tendency towards bigger clinics cannot be ascertained based on physical size, more clinics find themselves in need of secretaries or a receptionist. Co-habitation or sharing facilities with other kinds of health practitioners such as masseurs or physiotherapists is widespread and more than half the clinics have formally employed health practitioners other than chiropractors. This proportion has increased from 52 % in 2010 to 56 % in 2014.

Comparison with other countries in this respect must be looked upon with some reservation due to the considerable variation in the way related questions are posed. The Swiss survey from 2009 has 10 % of its respondents reporting to “practice in a multidisciplinary office” [10], and in The Netherlands the proportion of chiropractors who “shared facilities with providers from other health-care professions” was likewise 10 % in 2004. In Finland 14 % employed “a non-chiropractic assistant” according to the data from 2002 [5]. Only unpublished data from the Norwegian survey reveals a proportion of multidisciplinary setting close to the Danish level as 44 % of the clinics state to have at least one additional health practitioner at the clinic.

Despite the lack of homogeneity of the data, however, it seems safe to conclude that the co-habitation is far more developed in Denmark compared to most other countries, although further information on this subject as well as on the actual level of interdisciplinary collaboration is desirable.

### The chiropractic consultation

Looking at the average duration of a patient consultation, a remarkably stable pattern is revealed. Neither the first consultation with a new patient nor the follow-up consultations display any variation in length at all over the three surveys (40 min and 13 min respectively). In 2014 85 % of the chiropractors allotted 16–45 min to a new patient and approximately the same proportion allotted 6–15 min to follow-up consultations suggesting that Danish chiropractors have reached a rather firm concept of their individual treatment procedure.

The average duration of consultations does not differ very much across countries. In The Netherlands and Belgium an appointment with a new patient takes on average 41 and 36 min respectively, and follow-up consultations are done in 15 min in both countries [6, 7]. A comparison with Humphreys et al. suggests slightly longer consultations in Switzerland as new patients are seen 16–30 min by 23 % and 31–45 min by 60 % (Denmark: 33 and 52 %) and follow-up consultations are done in 6–10 min by 29 % and 11–15 min by 58 % (Denmark: 46 and 40 %). However very short first consultations with new patients of 15 min or less are not registered in Denmark but comprise 3 % of the Swiss consultations [10].

A similar pattern with comparatively long consultations is also seen in the other Nordic countries although different time categories make a one-on-one comparison impossible. In Norway 80 % dedicates 30–49 min to a new patient and 60 % see a follow-up patient for 15–20 min [9] while 80 % of the Finnish chiropractors allot 30–45 min to new patients and 15–30 min to follow-up patients respectively [5].

## Conclusions

This is the first study to describe the state and latest development of the chiropractic profession in Denmark using several consecutive surveys. Displaying various characteristics of both clinics and individual chiropractors, the image emerging is one of a stable profession where rapid or drastic changes are not taking place over short intervals of time.

The typical Danish chiropractor works in primary care, she is in her mid-forties with some 17 years of working experience. About half the profession graduated from University of Southern Denmark. The average clinic has 3–5 treatment rooms, is equipped with digital X-ray facilities and offers exercise facilities too. It employs at least one secretary, more than one chiropractor and, unlike clinics in our neighbouring countries, very often other health practitioners too. The average duration of the consultations, however, resembles that of other countries in the North-Western Europe, and it has been quite stable across all three surveys on 40 min for a new patient and 13 min for a follow-up consultation. Virtually all Danish chiropractors use manipulation as one of their treatment modalities.
